# Ultra-small aqueous glutathione-capped Ag–In–Se quantum dots: luminescence and vibrational properties†

**DOI:** 10.1039/d0ra07706b

**Published:** 2020-11-19

**Authors:** Oleksandra Raievska, Oleksandr Stroyuk, Volodymyr Dzhagan, Dmytro Solonenko, Dietrich R. T. Zahn

**Affiliations:** Semiconductor Physics, Chemnitz University of Technology Reichenhainer Straße 70 D-09107 Chemnitz Germany zahn@physik.tu-chemnitz.de; Center for Materials, Architectures, and Integration of Nanomembranes (MAIN), Chemnitz University of Technology D-09107 Chemnitz Germany; L. V. Pysarzhevsky Institute of Physical Chemistry, Nat. Acad. of Science of Ukraine 03028 Kyiv Ukraine; Forschungszentrum Jülich GmbH, Helmholtz-Institut Erlangen Nürnberg für Erneuerbare Energien (HI ERN) Immerwahrstr. 2 91058 Erlangen Germany o.stroyuk@fz-juelich.de alstroyuk@ukr.net; V. Lashkaryov Institute of Semiconductors Physics, National Academy of Sciences of Ukraine Kyiv Ukraine; Taras Shevchenko National University 01601 Kyiv Ukraine

## Abstract

We introduce a direct aqueous synthesis of luminescent 2–3 nm Ag–In–Se (AISe) quantum dots (QDs) capped by glutathione (GSH) complexes, where sodium selenosulfate Na_2_SeSO_3_ is used as a stable Se^2−^ precursor. A series of size-selected AISe QDs with distinctly different positions of absorption and PL bands can be separated from the original QD ensembles by using anti-solvent-induced size-selective precipitation. The AISe–GSH QDs emit broadband PL with the band maximum varying from 1.65 eV (750 nm) to 1.90 eV (650 nm) depending on the average QD size and composition. The PL quantum yield varies strongly with basic synthesis parameters (ratios of constituents, Zn addition, duration of thermal treatment, *etc.*) reaching 4% for “core” AISe and 12% for “core/shell” AISe/ZnS QDs. The shape and position of PL bands is interpreted in terms of the model of radiative recombination of a self-trapped exciton. The AISe–GSH QDs reveal phonon Raman spectra characteristic for small and Ag-deficient tetragonal Ag–In–Se QDs. The ability of ultra-small AISe QDs to support such “bulk-like” vibrations can be used for future deeper insights into structural and optical properties of this relatively new sort of QDs.

## Abstract 2

A direct aqueous synthesis, composition- and size-dependent absorption, photoluminescence, and vibrational properties of ultra-small glutathione-capped Ag-deficient Ag–In–Se quantum dots are reported.

## Graphical abstract

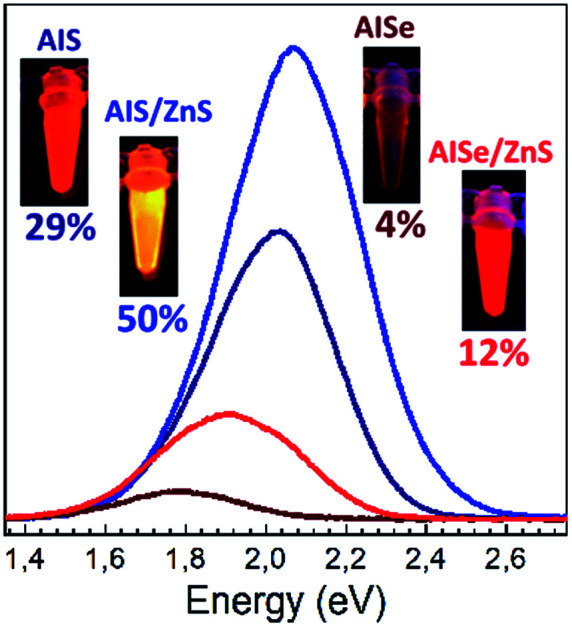

## Introduction

Multinary indium-based metal chalcogenide quantum dots (QDs), in particular, copper indium sulfide/selenide CuInS(Se)_2_ (CIS/CISe) and silver indium sulfide/selenide AgInS(Se)_2_ (AIS/AISe), have revealed a number of outstanding structural and optical properties, different from both binary metal–chalcogenide QDs (for example, CdS(Se) and PbS(Se)) and even from the corresponding bulk ternary compounds.^1–5^ Copper and silver indium chalcogenide QDs allow large deviations from stoichiometry, element substitutions, and doping. As a result, ternary QDs exhibit the same structure in a broad range of Cu(Ag) to In ratios (for example from 1 : 20 to 1 : 1), after substitution of Cu with Ag, In with Ga, and S with Se, as well as after heavy doping with metal cations.^1,3–6^ All these factors allow the position, structure, and intensity of absorption and photoluminescence (PL) bands of such “green”, eco-friendly QDs to be varied in a broad range.

In-based ternary QDs can emit strong PL which combines a broadband character and relatively large lifetimes (several hundred ns) with high intensity and distinct dependence on the QD size and composition. Recent efforts with AIS QDs allowed a PL quantum yield (PL QY) as high as 70% to be achieved.^2,3^ This initiated vivid discussions on the origins of their broadband PL indicating that even higher efficiencies can, in principle, be achieved.^1,2,4,6,7^

The combination of the broad variability of PL properties, high PL QYs, stability, and comparatively low toxicity of ternary QDs has inspired numerous studies aimed to shift the PL emission to the near-infrared (NIR) spectral range, that is, to the first “transparency window” of bio-tissues required, in particular, for *in vivo* luminescent biosensing applications.^2,8,9,11,12^ Reducing the bandgap of QD emitters by exchanging S for Se is apparently the most straightforward solution, however, the challenge of maintaining a reasonably high PL QY for the NIR-emitting CISe or AISe QDs has still to be addressed properly. On the other hand, the high absorption coefficients and the variability of the absorption band parameters of AISe QDs as well as the broad tunability of the positions of conduction band (CB) and valence band (VB) levels by variations in the composition/size of AISe QDs open bright perspectives for this light absorber as a photoactive component in solar cells,^11,13–18^ photoelectrochemical sensors,^19^ and photocatalytic systems.^20,21^

The most popular way towards luminescent AISe and Zn-doped AISe QDs is the incorporation of In^3+^ into primary Ag_2_Se particles that can be achieved by relatively high-temperature hot-injection syntheses in coordinating solvents (such as trioctylphosphine or oleylamine).^9,10,15,20,22–32^ Typically, Se^0^ is used as selenide precursor in these syntheses but sometimes rather exotic Se precursors are introduced to achieve fine control over the nucleation and growth of AISe QDs, such as bis(trimethylsilyl)selenide,^33^ cyclohexeno-selenodiazole,^21,30^ or Li[N(SeMe_3_)_2_].^34^ In the latter case, both composition and size of the AISe QDs can be independently varied resulting in “core” AISe and “core/shell” AISe/ZnSe QDs with a size from 2.4 nm to around 7 nm and three distinctly different compositions, Ag_3_In_5_Se_9_, AgIn_3_Se_5_, and AgIn_11_Se_17_, while a top PL QY of 73% is observed for Ag_3_In_5_Se_9_ cores with relatively thick ZnSe shells.^34^ Spontaneous reduction of Se^0^ by dodecanethiol yields a highly reactive selenium precursor allowing the hot-injection synthesis to be performed at relatively low temperature of 90 °C.^35^ In earlier heating-up syntheses single-molecule precursors with a pre-defined Ag-to-In ratio (such as (PPh_3_)_2_AgIn(SeC(O)Ph)_4_, Ph – C_6_H_5_) were used to control the composition of final AISe QDs.^36,37^

After the synthesis, such AISe QDs can be transferred into aqueous solutions by ligand exchange procedures^16,17,20,27,32^ or by micellar solubilization.^9,23^ In this way, stable aqueous AISe QDs with PL maxima tailored from 700 nm to 820 nm and PL QYs of 40–50% were produced using mercaptopropionic acid (MPA)^16,17,20,27^ or octylamine-modified polyacrylic acid^23^ as phase-transfer agents.

In contrast to the hot-injection procedure, direct aqueous synthesis of AISe QDs was found to be rather challenging in view of the limited scope or instability of selenide precursors that can be applied in such systems or because of a limited control over the formation of QDs when stable selenide precursors are reduced to Se^2−^ by strong reductants. For example, core/shell AISe/ZnSe QDs with a core size of 3.3 nm and a PL QY of up to 31% were produced directly in aqueous solutions using MPA as a capping ligand and NaHSe as a selenium precursor.^38^ The latter can be readily oxidized to Se^0^ by O_2_ and, therefore, the synthesis should be performed in an inert atmosphere.

When stable selenide precursors such as SeO_2_,^19^ Na_2_SeO_3_ (ref. 39) or Se^0^ (ref. 1, 13 and 40) are used, the introduction of a strong reductant is needed, typically NaBH_4_, to convert selenium from a deeper oxidation state to Se^2−^*in situ* and form AISe QDs.^11,39^ In such conditions the process of QD growth is only partially controlled and the PL QYs achieved by this approach are typically very low (for core AISe QDs).^11,39^ By combining chelating capacities of thioglycolic acid (TGA) and gelatin as stabilizers, aqueous NIR-emitting core–shell AISe/ZnS colloidal QDs with PL QYs of 10–20% were produced.^40^ Another option is the recently reported electrochemical *in situ* reduction of selenium to Se^2−^ which then reacts with Ag^I^–GSH and In^III^–GSH complexes.^41^ This approach is very promising as it yields 3–5 nm QDs with the PL QY reaching almost 20% at an optimal Ag-to-In ratio of around 1 : 5. A similar electrodeposition method with SeO_2_ as a selenide precursor and no capping ligands were applied to sensitize TiO_2_ films with relatively large (6–8 nm) non-luminescent AISe particles.^18^

Earlier we reported on the aqueous syntheses of non-stoichiometric CIS QDs,^42^ AIS QDs,^43–45^ copper-doped AIS QDs,^46^ and alloyed (Cu,Ag)–In–S QDs^47^ stabilized by complexes of surface cations with TGA^42,43,46^ and glutathione (GSH).^44,45,47^ Such QDs exhibit broadband PL in the visible spectral range with PL QYs reaching 50% for AIS–GSH QDs.^44^ The next logical step in these studies was towards shifting the position of the PL band, which is typically centered at around 600 nm for AIS–GSH QDs, to the NIR range by switching from sulfide to selenide. This attempt was backed up by our previous experience with aqueous syntheses of CdSe QDs with selenide anions released *in situ* either *via* the reduction of selenite anions by a stabilizing ligand (TGA)^48,49^ or *via* intramolecular decomposition of sodium selenosulfate.^50,51^

The first approach appeared to be unsuccessful when applied for the synthesis of GSH-capped AISe QDs, most probably, due to much lower reducing capacity of GSH as compared to TGA and a tighter binding of Ag^+^ and In^3+^ cations by the GSH ligand. However, the second approach was found to be quite efficient, allowing stable and luminescent AISe QDs to be produced in aqueous solutions in the course of a mild thermal treatment of alkaline solutions containing Ag^I^–GSH and In^III^–GSH complexes and SeSO_3_^2−^ anions. In this way, AISe QDs are formed with no additional strong reductants (like NaBH_4_ or external current) with no release of selenium-containing volatile or unstable byproducts and in the presence of ambient air. The precursor itself (Na_2_SeSO_3_) is rather stable and can only release Se^2−^ species during the QD synthesis while they are bound by metal cations. We note that sodium selenosulfate has not yet been reported as a selenide source for the aqueous synthesis of small (2–3 nm) and luminescent AISe QDs.

As in the previous case of AIS–GSH QDs, the selenosulfate-based synthesis is flexible and allows the composition and size of QDs to be varied as well as post-synthesis size-selection and ZnS shell deposition procedures to be performed. In the present paper we discuss the most efficient ways of affecting the composition, size, and crystallinity of GSH-capped AISe QDs as well as special features that can be observed in their light absorption and emission as well as vibrational Raman spectra. Besides providing the vibrational (phonon) spectra themselves, resonant Raman spectroscopy is an efficient tool of probing both the lattice structure of semiconductor QDs and local electronic resonances related either with intentional heterogeneity, like in core/shell structures,^52^ or spontaneous formation of secondary phases,^53^ common in complex chalcogenide QDs but hardly detectable by other structural techniques.^54^ Based on our previous findings of distinct vibrational patterns of aqueous II–VI^55^ and M–In–S QDs^56^ of ultrasmall size, an investigation of similar small Ag–In–Se is additionally motivated.

## Materials and methods

### Materials

InCl_3_, AgNO_3_, reduced glutathione, aqueous 5.0 M NH_4_OH solution, 69 w.% HNO_3_, citric acid, Se powder, Na_2_SO_3_, Zn(CH_3_COO)_2_·2H_2_O, and 2-propanol were supplied by Merk and used without additional purification. Deionised (DI) water was used in all experiments. The stock solution of sodium selenosulfate (SS) was prepared by reacting selenium powder with sodium sulfite in a hot aqueous solution. For this aim, 12.7 g Na_2_SO_3_ was dissolved in 100 mL DI water and 1.58 g fine Se powder added to this solution. The mixture was then boiled with a reflux condenser for 3 h till complete dissolution of the Se powder. The final SS solution was kept at 5–7 °C in the dark. Warning: sodium selenosulfate can potentially be toxic if digested or applied to unprotected skin. The stock solution of NH_4_OH should be kept thoroughly closed to maintain the concentration of highly volatile ammonia constant.

### Synthesis of AISe QDs

Silver–indium–selenide QDs were produced by reacting a mixture of Ag(i) and In(iii) GSH complexes with selenide anions evolving at the decomposition of sodium selenosulfate Na_2_SeSO_3_. In a typical procedure, 0.85 mL DI water was mixed with 1.2 mL aqueous 0.5 M GSH solution, and 0.4 mL aqueous 1.0 M InCl_3_ solution (containing 0.25 M HNO_3_ to avoid In^3+^ hydrolysis). The mixture was stirred for 2 min to allow In(iii)–GSH complexes to be formed in an equilibrium and then 0.5 mL aqueous 5.0 M NH_4_OH solution was added and the resulting mixture stirred for 5 min till complete dissolution of the precipitate. This was followed by subsequental addition of 0.05 mL aqueous 2.0 M solution of citric acid, 1.0 mL aqueous 0.1 M solution of AgNO_3_, and 1.0 mL aqueous 0.8 M solution of Na_2_SO_3_. The mixture was stirred for 2 min and then 2.5 mL aqueous 0.2 M stock solution of Na_2_SeSO_3_ (containing 0.8 M Na_2_SO_3_) and after 2 min refluxing 2.5 mL aqueous 0.5 M GSH solution were added with the total volume of the reacting mixture reaching 10.0 mL. The molar concentrations of principal components in the final mixture were [SS] = 0.05 M, [AgNO_3_] = 0.01 M, and [InCl_3_] = 0.04 M.

The mixture was subjected to heating at 96–98 °C (in cylindric vials 1 cm in diameter with no reflux in a boiling water bath) for *t*_H_ = 45–60 min. The synthesis was finalized by QD purification *via* precipitation. At that, 0.1 mL aqueous 5.0 M NH_4_OH solution was added to 10.0 mL of AISe colloid and the solution was mixed with 10.0 mL 2-propanol (water : 2-propanol = 1 : 1 v/v) and subjected to centrifugation at room temperature and a rate of around 5000 rpm. The viscous precipitate was separated from the supernatant and redissolved DI water (water is added till the total volume of 1.0 mL is reached) resulting in a 10-fold concentration of the colloidal solution with respect to the original non-purified solution. The nominal molar Ag(i) concentration in the final concentrated solution was 0.1 M.

To produce Zn-diffused AISe QDs (ZAISe) 0.5 mL aqueous 1.0 M Zn(ii) acetate solution was added to 10 mL of non-purified AISe cores and the colloidal solution heated for additional 15 min. In the case of ZAISe, the purification was performed by adding first 0.1 mL aqueous 5.0 M NH_4_OH solution followed by mixing with 2-propanol (1 : 1 v/v), centrifugation, and redispersion of the precipitate in 1.0 mL DI water (water is added till the total volume of 1.0 mL is reached).

### Optimization of the synthesis

The protocol of the synthesis of AISe (ZAISe) QDs was optimized to produce the most stable and luminescent QDs by varying the principal parameters of the synthesis, namely, the molar ratios of silver(i) to indium(iii), silver(i) to zinc(ii), and silver(i) to selenosulfate (SS), as well as the duration of post-synthetic heat treatment at 96–98 °C. In particular, the Ag^+^ concentration was varied from 0 to 0.0175 M ([SS] = 0.05 M, [In^3+^] = 0.04 M, *t*_H_ = 120 min); the SS concentration was changed from 0.03 M to 1.0 M ([Ag^+^] = 0.01 M, [In^3+^] = 0.04 M, *t*_H_ = 120 min); *t*_H_ was varied from 0 to 240 min ([SS] = 0.05 M, [Ag^+^] = 0.01 M, [In^3+^] = 0.04 M); the Zn^2+^ concentration was increased from 0.0075 M to 0.11 M ([SS] = 0.05 M, [Ag^+^] = 0.01 M, [In^3+^] = 0.04 M, *t*_H_ = 120 min). The GSH concentration was kept constant at 0.185 M.

### Size selection procedure

The size selection was performed using the well-established method of size-selective precipitation adapted for aqueous ternary QDs as reported by us earlier^43,44^ by using 2-propanol as an anti-solvent. In a typical procedure, 10 mL of colloidal AISe solution ([SS] = 0.05 M, [Ag^+^] = 0.01 M, [In^3+^] = 0.04 M, [GSH] = 0.185 M, *t*_H_ = 60 min) was cooled to room temperature, and 0.1 mL aqueous 5.0 M NH_4_OH solution was added prior the fractionation. Then, 2-propanol was added portion-wise by 1.0 mL steps followed by stirring of the mixture. The first batch of precipitate formed after the addition of 4.0 mL 2-propanol was separated from the supernatant and redispersed in 1.0 mL DI water (fraction 1). The next fraction was produced by adding 1.0 mL 2-propanol to the supernatant which contains already 4.0 mL 2-propanol and separating the precipitate (fraction 2). The latter procedure was repeated several times by adding 1.0 mL 2-propanol and increasing the total anti-solvent content to 6.0 mL (fraction 3), 7.0 mL (fraction 4), 8.0 mL (fraction 5), and 9.0 mL (fraction 6). Fractions 7 and 8 produced with 10.0 and 11.0 mL 2-propanol contained negligible amounts of the viscous precipitate and showed no coloration.

### Instruments

Absorption and PL spectra were registered in standard 10.0 mm quartz optical cuvettes using a Black Comet CXR-SR UV/Vis/NIR spectrometer (StellarNet Inc., USA) equipped with miniature deuterium/halogene lamps (absorption spectra) or a 390 nm diode (PL spectra) as excitation sources and 100 μm slits in the range of 220–1100 nm. Colloidal solutions were diluted prior to the measurements (1.0 μL per 2.5 mL DI water). Absorption spectra were acquired at an accumulation time of 100 ms with five consecutively taken spectra averaged. The absorbance of a cuvette with pure DI water was substracted from the final spectrum. PL spectra were registered with an acquisition time of 1000 ms and normalized to the absorbance at 390 nm. The relative PL quantum yield (QY) of AISe and AISe/ZnS QDs was evaluated using previously reported aqueous GSH-capped AIS/ZnS QDs^44,45^ as a luminescent standard with an absolute PL QY of 50%.

Raman spectra were registered using a Horiba LabRAM HR800 spectrometer equipped with a cooled CCD camera. The excitation was provided by a solid-state lasers (*λ*_exc_ = 488 nm and 514.7 nm). The instrumental resolution was better than 2 cm^−1^. The samples were drop-casted on cleaned silicon wafers and dried in vacuum. Before the deposition, MV^2+^ was added to the QD colloid in an equimolar amount with respect to the total Ag^I^ concentration to quench emission as reported earlier.^47,52^ The Si phonon peak at 520 cm^−1^ served as internal calibration of the Raman peak positions.

X-ray diffractograms were collected using a Rigaku SmartLab diffractometer in an angle range of 2*θ* = 5–100° with a step rate of 0.05° per min using 9 kV copper *K*_α_ irradiation. The samples were produced by drop-casting QD colloids on a glass plate at room temperature. The drop-cast solutions were dried in vacuum. Equal amounts of the various colloidal samples were deposited on the glass plates to enable comparison of the peak intensities.

Atomic Force Microscopy (AFM) images were acquired with an AFM 5500 from Keysight (Agilent). The AFM tip has a radius of 10 nm and the Si cantilever a resonance frequency of 180 kHz. The samples were prepared by drop-casting of a very diluted (in the order of 10^−5^ M in terms of Ag^I^ concentration) colloids on a freshly cleaved mica surface and dried in a nitrogen stream at room temperature.

The QD size distribution charts were plotted based on AFM measurements of the height profiles for several hundred separate QDs. For this aim, the AFM images were pre-processed using the Gwyddion package (with “plane substraction” and “aligning rows” filters and zero fixing). The pre-processed images were marked by using the Gwyddion threshold-based grain marking tool with slope, curvature, and height parameters set respectively to 50, 100, and *ca.* 30%. The height distribution was then produced using marked images and a standard Gwyddion tool with median height as an abscissa parameter and fitted with a Gaussian function to determine the average size and size distribution of AISe QDs on a mica surface.

X-ray photoelectron spectroscopic (XPS) measurements were performed with an ESCALAB 250Xi X-ray Photoelectron Spectrometer Microprobe (Thermo Scientific) equipped with a monochromatic Al *K*_α_ (1486.86 eV) X-ray source. A pass energy of 200 eV was used for survey spectra and 20 eV for high-resolution core-level spectra (providing a spectral resolution of 0.5 eV). Spectra deconvolution and quantification were performed using the Avantage Data System (Thermo Scientific). To prevent charging during XPS measurement, the samples were flooded with low kinetic energy electrons and Ar^+^ ions. Finally, the spectra were corrected to C 1s peak at 284.8 eV as the common internal standard for binding energy (BE) calibration.

## Results and discussion

Sodium selenosulfate is a very convenient source of selenide anions in the synthesis of metal selenide QDs as it is relatively stable and converts to Se^2−^*in situ* and only under boiling in the presence of metal cations (or complexes) allowing the course of the reaction to be controlled in a precise manner. A 4-fold excess of sodium sulfite necessary for the transformation of metallic selenium into SS efficiently and quantitatively reduces dissolved oxygen allowing the synthesis to be performed in a quasi “inert” atmosphere without argon or nitrogen purging through the solution.^50,51^ Additionally, excessive sulfite can readily reduce any Se^0^ species that can form at the colloid/air interface during the synthesis and during the thermal treatment allowing the synthesis to be optimized to completely avoid the formation of colloidal Se^0^ by-products or Se^0^ species or other oxidized forms of selenium on the surface of metal selenide QDs.

In this section we discuss the optimization of the synthetic protocol to achieve the highest PL QY through variations of the nominal (set at the synthesis) ratio of Ag^I^ to In^III^, duration of the thermal treatment *t*_H_, and ratios of SS to Ag^I^, and Zn^II^ to Ag^I^, as well as size-dependent optical characteristics of AISe QDs produced by the size-selective precipitation/redispersion. At that, the structure/morphology of AISe QDs is probed by a combination of XRD, XPS, and AFM. We also present a survey of vibrational characteristics of AISe QDs probed by Raman spectroscopy at room and low temperatures.

### Variation of Ag-to-In ratio

It was found that an increase in the silver content in non-stoichiometric AISe QDs results in a gradual shift of the absorption threshold of colloidal solutions to longer wavelengths with the color of the solutions developing from light orange to dark red.

**Fig. 1:**
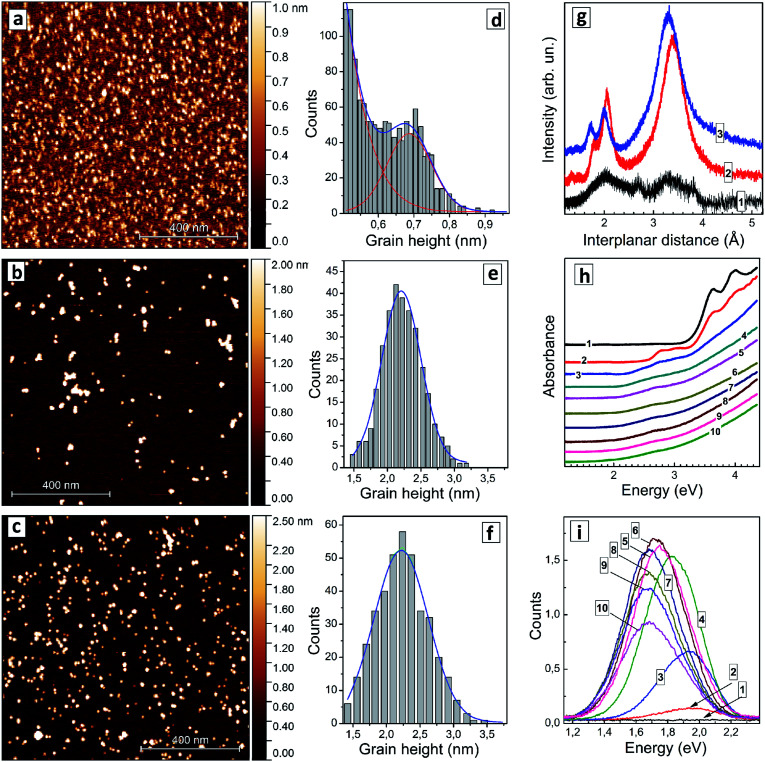
(a–f) AFM images (a–c) and grain height distributions (d–f) of AISe QDs produced at nominal [In]/[Ag] ratio of 10 (a and d), 4 (b and e), and 2.7 (c and f). (g) XRD patterns of AISe QDs produced at [In]/[Ag] = 10 (curve 1) and 4 (curve 2). Curve 3 represents XRD pattern of Ag–In–S QDs produced at [In]/[Ag] = 4. (h and i) absorption (h) and PL spectra (k) of AISe QDs produced at [In]/[Ag] = 0 (curves 1), 20 (2), 10 (3), 6.7 (4), 5 (5), 4.4 (6), 4 (7), 3.6 (8), 3.3 (9), and 2.7 (10).

The colloidal AISe particles can be readily dispersed on freshly cleaved mica, allowing size distributions of relatively large ensembles of single QDs to be determined *via* AFM. This method enables a simultaneous characterization of several hundreds of QDs. Fig. 1 shows a set of AFM images (a–c) and corresponding size distributions (d–f) of AISe QDs with three different Ag-to-In ratios. The sample with [In]/[Ag] = 10 reveals an ensemble of nanoscale particles with the size smaller than 0.5 nm overlapping with another ensemble with an average size of 0.7 nm (Fig. 1a and d). Only the first ensemble was detected for samples synthesized in the absence of silver, suggesting that the ensemble of smaller particles can be ascribed to In-related clusters while the second ensemble with an average size of 0.7 nm represents, most probably, Ag–In–Se nuclei stabilized by GSH ligands. As the [In]/[Ag] ratio is decreased to 4 the particles grow to 2.2–2.3 nm (Fig. 1b and e), the size remaining at this value for AISe QDs with a higher Ag content ([In]/[Ag] = 2.7, Fig. 1c and f).

An XRD pattern of particles produced at [In]/[Ag] = 10 (Fig. 1g, curve 1) shows only broad peaks indicating the amorphous character of such particles, which are, most probably, silver-doped polynuclear complexes of In(iii) with GSH. As the [In]/[Ag] ratio is decreased to 4 a distinct motif of tetragonal chalcopyrite Ag–In–Se^11^ can be observed (Fig. 1g, curve 2) with interplanar (112), (220), and (312) distances of 3.39 Å, 2.05 Å, and 1.80 Å, respectively, close to the lattice parameters of stoichiometric AgInSe_2_ (ref. 10, 11 and 29) and AgIn_5_Se_8_.^10^ An identical XRD pattern was also registered for AISe QDs synthesized at [In]/[Ag] = 2.7 (not shown). The average size of AISe QDs evaluated from the broadening of XRD reflections using the Scherrer equation was found in both cases to be around 2 nm, in accordance with the AFM observations. The diffractogram of AISe QDs differs distinctly from the XRD pattern of Ag–In–S QDs with [In]/[Ag] = 4 (Fig. 1g, curve 3) reported by us earlier^44^ with corresponding lattice parameters of 3.31 Å, 2.00 Å, and 1.73 Å.

The absorption spectrum of the colloidal solution produced without silver (Fig. 1h, curve 1) shows several absorption peaks in the spectral range above 3 eV. The most prominent ones are at 4.0 eV (310 nm), 3.95 eV (314 nm), and 3.62 eV (343 nm). These peaks can be tentatively attributed to molecular species, such as complexes of In(iii) with GSH or indium selenide clusters capped with GSH. As silver is introduced, at [In]/[Ag] = 20 (Fig. 1h, curve 2), two additional peaks appear at around 3.10 eV (400 nm) and at 2.78 eV (446 nm) indicating the formation of new species, most probably, AISe clusters stabilized by GSH ligands. This assignment is supported by AFM data showing the presence of clusters with a size of around 0.7 nm, which can be characterized by the new two absorption peaks. As the [In]/[Ag] ratio is decreased to 10 (Fig. 1h, curve 3) both peaks get smeared and shift to lower energies.

At higher Ag contents only a broad peak at around 2.6–2.7 eV can be distinguished, while the absorption “tail” extends into the visible range with no distinct edge or other prominent features. As shown below in more detail on other examples such structure of the absorption band of AISe–GSH QDs does not allow precise positions of both absorption band edge and absorption maximum at higher energies to be extracted from the absorption spectra. The presentation of absorption spectra in coordinates of the Tauc equation typically does not provide linear sections long enough for the band gap to be determined with satisfactory precision. Similarly broad absorption bands were detected also for a size-selected series of AISe QDs (discussed below) indicating that the absorption edge is smeared not by a broad QD size distribution but rather by Urbach tails inherent to such ternary non-stoichiometric QDs.^6^

The absorption maximum is broad and of low intensity introducing a high uncertainty as well. In this view, the values of the bandgap evaluated from Tauc plots (*E*^T^_g_) as well as the positions of absorption maxima (*E*_max_) presented below and in the ESI† should be regarded as approximate values.

Both *E*^T^_g_ and *E*_max_ were found to decrease as the silver content grew, from *E*^T^_g_ = 2.15 eV and *E*_max_ = 2.72 eV for [In]/[Ag] = 10 to *E*^T^_g_ = 1.72 eV and *E*_max_ = 2.63 eV for [In]/[Ag] = 2.7 (ESI, Table S1†). These changes can be assigned to a growing contribution of Ag^+^ d-orbitals to the conduction band (CB) of AISe QDs resulting in a bandgap narrowing, similarly as it was observed earlier for AIS QDs^6,43,44^ as well as to possible variations of the average size of AISe QDs produced at different [In]/[Ag] ratios as discussed above.

The AISe QDs emit broadband PL with the band intensity and position depending on the Ag-to-In ratio significantly. At the same time, no PL can be detected for the samples prepared in the absence of Ag^+^ (Fig. 1i). The shape of the PL band can in all cases be described by a single Gaussian profile (ESI, Fig. S1a†) with the full width at half maximum (FWHM_PL_) remaining roughly constant between 340 and 360 meV (ESI, Table S1†).

The PL band maximum *E*_PL_ shifts from 1.94 eV (640 nm) for [In]/[Ag] = 20 to 1.72 eV (720 nm) for [In]/[Ag] = 2.7–3.3 (ESI, Table S1, Fig. S1b†) simultaneously with *E*^T^_g_ and *E*_max_. A similar trend was also reported earlier for composition-selected aqueous GSH-capped Zn-doped AISe QDs.^12^ The PL intensity was found to grow drastically as the [In]/[Ag] ratio was decreased from 20 to 6, reaching a plateau at 4–4.4 and decreasing steeply at higher silver contents (Fig. 1i; ESI, Fig. S1c, Table S1†). Based on these observations we choose the silver-to-indium ratio at [In]/[Ag] = 4 for further optimization of the synthetic protocol.

### Variation of thermal treatment duration

The duration of post-synthesis heating (*t*_H_) performed close to the boiling point of aqueous colloidal AISe solutions is a vital step which strongly affects both the optical properties and the stability of AISe QDs. Relatively low PL intensity and a tendency to agglomeration are observed both at short and at long thermal treatments indicating the importance of optimization of this parameter as well as more in-depth insights into the origins of changes in emissive capability.

**Fig. 2:**
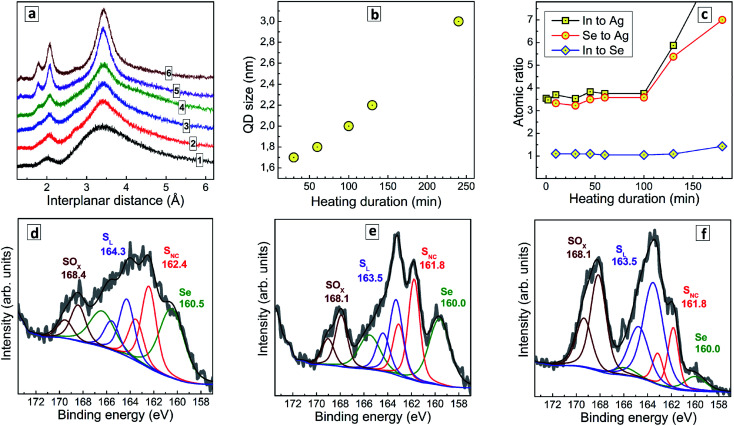
(a) XRD patterns of AISe QDs produced with no thermal treatment (curve 1) and heating at 96–98 °C for 30 min (curve 2), 60 min (3), 100 min (4), 130 min (5), and 240 min (6). (b) Average size of AISe QDs calculated from XRD patterns as a function of the heat treatment duration. (c) Atomic ratios of In to Ag (squares), Se to Ag (circles), and In to Se (diamonds) derived from XPS data as a function of the heat treatment duration of AISe QDs. (d–f) High resolution X-ray photoelectron spectra in the range of S 2p/Se 3p binding energies for unheated AISe QDs (d) as well as for the QDs heated during 30 min (e) and 130 min (f). the Se to S_NC_ ratio is 1.1 (d), 0.99 (e) and 1.02 (f). Multicolored lines represent the best fit of the experimental spectra with combinations of Gauss profiles, black is the total envelope profile, blue is the baseline.

When no thermal treatment is applied, the XRD pattern of AISe QDs shows strongly broadened peaks on the position expected for the (112), (220), and (312) reflections (Fig. 2a, curve 1) indicating a predominantly amorphous nature of such QDs. As the thermal treatment is applied and its duration increased, the characteristic (112), (220), and (312) reflections become more pronounced and narrow, while their positions remain roughly the same (curves 2–6). This fact, along with a growth of the intensity ratio of higher-order reflections to the intensity of (112) peak indicate an extensive crystallization of AISe QDs at longer annealing times. Estimations made using Scherrer equation showed that the average QD size increases monotonously from 1.9 nm for *t*_H_ = 30 min up to 3.0 nm for *t*_H_ = 240 min (Fig. 2b).

To gain an insight into the transformations of the QDs composition during the thermal treatment we performed a XPS study of samples produced at a different *t*_H_. Survey X-ray photoelectron spectra (ESI, Fig. S2†) show the same elemental composition for all studied samples, with the obvious presence of Ag, In, Se, S and N (from GSH), O (both from GSH and bound water), C, and Na (most probably, as Na–GSH salt). High-resolution spectra of Ag 3d, In 3d, and Se 3d for a representative sample prepared at *t*_H_ = 60 min (ESI, Fig. S3†) show these elements to be present in the expected oxidation states (Ag^+^, In^3+^, Se^2−^). The C 1s, N 1s, and O 1s series (ESI, Fig. S3†) can be interpreted in terms of the presence of GSH ligand and bound water.

Fig. 2c illustrates the evolution of atomic In-to-Ag, Se-to-Ag, and In-to-Se ratios as a function of the thermal treatment duration. The samples show rather constant values of all three ratios during the first 100 min of heating, but then the QD growth mode changes and additional quantities of indium selenide start to deposit on the AISe QDs as evidenced by growing In-to-Ag and Se-to-Ag ratios at a rather stable In-to-Se ratio. This observation is indirectly corroborated by a deteriorated stability of AISe colloids treated longer than 100 min. In contrast to the colloidal systems produced at *t*_H_ < 100 min which are rather stable and can be stored for many months at 5–7 °C, the AISe QDs synthesized at *t*_H_ > 100 min precipitate from both for non-purified and purified solutions within 24 h after the heat treatment.

Simultaneously with the changes in the In-to-Ag and Se-to-Ag ratios we observe the formation of a layer of oxidized sulfurous products on the surface of AISe QDs as evidenced by the analysis of S 2p/Se 3p series (Fig. 2d–f). The structure of this spectral section is similar for all samples studied; however, the relative contribution of the components differs quite drastically. In particular, the non-heated AISe QDs show a doublet at 160.5/166.5 typical for Se^2−^, and three sulfur-related doublets with the 2p_3/2_ peak observed, respectively, at 162.4 eV, 164.3 eV, and 168.4 eV (Fig. 2d). These components can be assigned to sulfide ions in a metal–sulfide lattice, S_NC_, to sulfur from GSH ligand, S_L_, and to products of sulfur oxidation by ambient air or sulfite residuals, SO_*x*_, respectively. As the duration of heat treatment is increased, the SO_*x*_ component grows and becomes a dominant sulfur species for the sample produced at *t*_H_ = 130 min (Fig. 2f), while the spectral contribution of Se-related signals decreases considerably.

It should also be noted that the ratio of integral intensities of Se 3p_3/2_ to S_NC_ 2p_3/2_ peaks remains almost constant, namely 1.10, 0.99, and 1.02 for *t*_H_ = 0, 30 min, and 130 min, respectively, indicating that no AIS shell forms on the AISe core during the thermal treatment. Taking together these facts allows us to conclude that heating results in a growth of AISe QDs accompanied by oxidation of surface GSH ligand and formation of an indium selenide shell (most probably from the residuals adsorbed on the QD surface). The reason for not observing the oxidation of the ligand at shorter *t*_H_ is most probably the presence of an excess of Na_2_SO_3_ which is introduced together with SS and protects the AISe–GSH QDs from oxidation *via* efficient reduction of molecular oxygen, which is a chain process and can rapidly consume the entire Na_2_SO_3_ present in the solution and leave QDs unprotected against oxidation at *t*_H_ > 100 min.

**Fig. 3:**
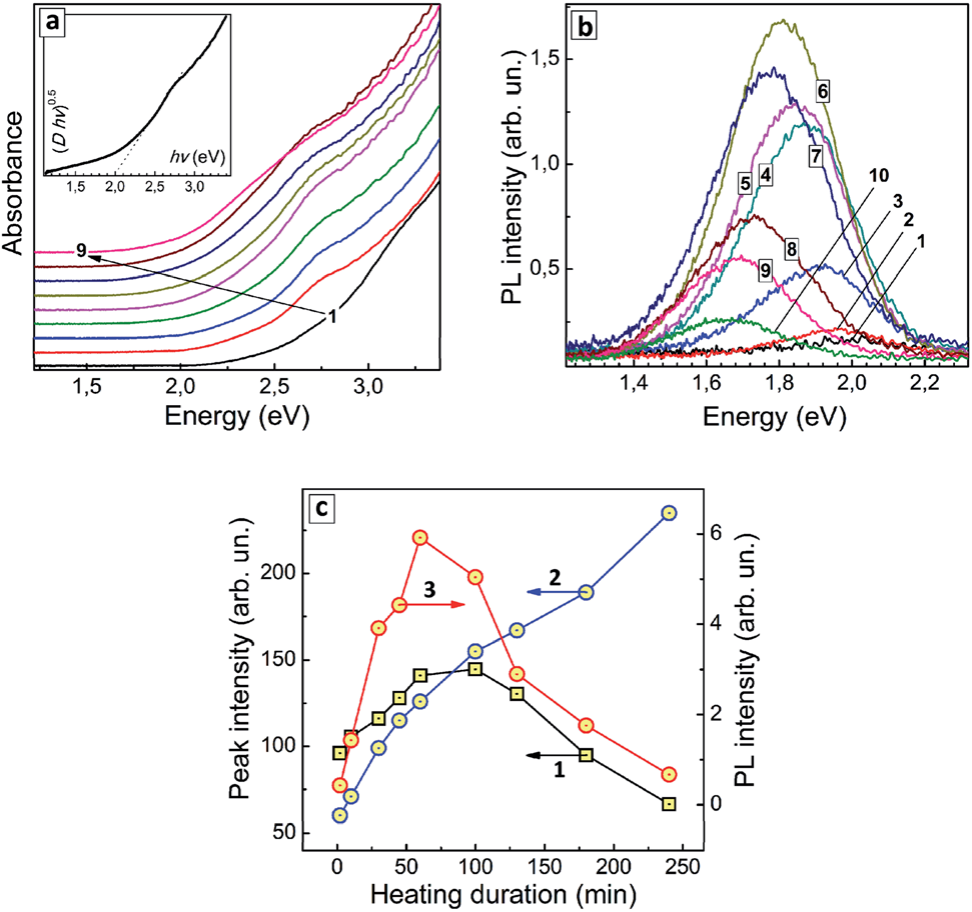
(a and b) Absorption spectra (a) and PL spectra (b) of AISe QDs produced with a different duration of thermal treatment – 0 min (curves 1), 2 min (2), 10 min (3), 30 min (4), 45 min (5), 60 min (6), 100 min (7), 130 min (8), 180 min (9), and 240 min (10). Insert in (a): absorption spectrum of the sample treated for 30 min presented as “(*D* × *hv*)^0.5^*vs. hv*”, where *D* is optical density. (c) Intensities of absorption components peaked at around 2.75 eV (squares 1) and around 2.45 eV (circles 2), as well as total PL intensity (circles 3) as functions of the thermal treatment duration. Lines connecting the data points are guides for the eye only.

The thermal treatment of AISe QDs results in the formation of a distinct spectral maximum at around 2.75 eV (around 450 nm) and a gradual shift of the absorption band edge toward lower energies (Fig. 3a). As XPS data showed no changes in the composition of the AIS QDs (at least for the first 100 min of treatment) the observed spectral changes can be associated exclusively with variations in the QD size/size distribution.

An exemplary spectrum plotted in Fig. 3a (inset) in the coordinates of the Tauc equation shows that no precise determination of the bandgaps can be performed, because, as discussed above, the linear section of the spectral curve is not long enough. We could expect both a narrowing of the size distribution and defect healing to occur during the heat treatment with both these factors assumingly resulting in a more distinct absorption edge and lower Urbach tail. On the contrary, we observe the same featureless structure of the absorption spectra for all samples in this series indicating an inherent character of such spectral shape. To circumvent this obstacle we analyzed the heating-induced spectral changes by subtracting the absorption spectrum for *t*_H_ = 0 (curve 1 in Fig. 3a) from all other spectra in the series. The resulting differential spectra (ESI, Fig. S4a†) reveal a line shape that can be well fitted with two Gaussian profiles, with maxima at around 2.75 eV and 2.45 eV (ESI, Fig. S4b and c†).

As the duration of thermal treatment is increased, an apparent intensity redistribution of these peaks is observed, with the lower-energy band becoming more pronounced at the expense of the higher-energy component gradually diminishing (compare Fig. S4b and c, in ESI†). This tendency is exemplified in Fig. 3c (curves 1 and 2) showing that the lower-energy absorption feature increases monotonously with *t*_H_, while the higher-energy feature passes through a maximum at *t*_H_ = 60–100 min and decreases at longer heating times. It should be explicitly stated that at present state of our understanding we cannot associate a particular structural species or types of electron transitions with these two spectral components. Nevertheless, they demonstrate two unambiguous tendencies during the heat treatment – (i) the gradual shift of the absorption edge to lower energies and the growth of longer-wavelength absorption (2.45 eV component) and (ii) the formation of QD species with the corresponding excitonic maximum in the absorption spectra (2.75 eV component), as well as their gradual extinction due to Ostwald ripening during longer heat treatment (*t*_H_ > 100 min).

As shown above by the XPS results, the thermal treatment results in a gradual deposition of an indium selenide shell which can also contribute to the lower-energy absorption maximum. The XRD data showed an increase in the average size of AISe QDs during the heat treatment with the positions of the reflections being roughly the same. To unite XPS and XRD data we need to assume that the indium selenide shell is amorphous and cannot be detected by XRD. At the same time, it can contribute to absorption, in particular, to the growth of the lower-energy maximum, along with the increase of the AISe QD size and affect the efficiency of PL emission both by light shielding effect and by introducing new recombination centers on the QD surface.

Similar to the absorption spectra, the PL spectra of AISe QDs transform during the thermal treatment, with the PL maximum shifting to lower energies (from 1.99 eV/624 nm at *t*_H_ = 0 to 1.66 eV/748 nm at *t*_H_ = 240 min, see ESI, Table S2†). The PL intensity is also a function of *t*_H_, increasing by almost 30 times during the first 60 min of thermal treatment and decreasing gradually at higher *t*_H_ values (Fig. 3b; ESI, Table S2†).

The dependence of integral PL intensity of AISe–GSH QDs on *t*_H_ (Fig. 3c, curve 3) shows a prominent similarity to the dependence of the intensity of the 2.75 eV absorption peak on the treatment duration (curve 2). The PL intensity reaches highest values roughly in the same *t*_H_ range as the intensity of the higher-energy absorption component allowing to conclude that the PL emission is associated with the presence of AISe QDs that exhibit the excitonic maxima at around 2.7 eV.

Summarizing the above discussed features of AISe QDs introduced by the post-synthesis thermal treatment we may assume two factors as possible reasons for the observed dependence of PL intensity on *t*_H_: (i) an increase in the probability of the radiative recombination at the beginning of thermal treatment due to more extensive crystallization of the initially amorphous QDs towards highly-emitting QDs featuring an excitonic peak at around 2.7 eV; (ii) a decrease in the efficiency of the radiative recombination at longer *t*_H_ due to the conversion of such highly-emitting QDs into larger non-emissive QDs, formation of an amorphous indium selenide shell as well as partial oxidation of the surface ligands, the latter factors resulting, most probably, in new surface states responsible for the non-radiative recombination. These two competing tendencies result in an optimal range of *t*_H_, from which *t*_H_ = 60 min was selected as optimal for the synthesis of the most luminescent AISe QDs.

It should also be noted that the spectral width of PL bands does not show any considerable change during the thermal treatment, fluctuating insignificantly around 300 meV (ESI, Table S2†). In this view, AISe QDs are similar to the previously studied case of AIS QDs, for which FWHM_PL_ remains stable at various variations of QD size, thermal treatment duration, ZnS shell formation, and variations of ambient temperature.^45^ Such behavior is indicative of the self-trapped exciton recombination as a mechanism of PL emission, when the spectral width of the PL band is defined not by distributions of QD size and/or energy of defects (which are expected to be strongly affected by the thermal treatment), but by the strength of electron–phonon interaction and by the energy of purely electronic band states of the QDs.

### SS to Ag ratio

In our earlier reports^50,51^ we observed a rather strong dependence of the growth dynamics of CdSe QDs in aqueous solutions on the ratio of CdCl_2_ and Na_2_SeSO_3_ associated with differences in primary nucleation rate at different rates of Se^2−^ release from SS as well as from the possibility of the formation of selenosulfate complexes of Cd(ii) that can affect the rate of SS anion decomposition and monomer CdSe concentration. In view of our previous result, we may expect a considerable influence of Ag-to-SS ratio on both the composition and the optical properties of AISe QDs to be observed as well.

**Fig. 4:**
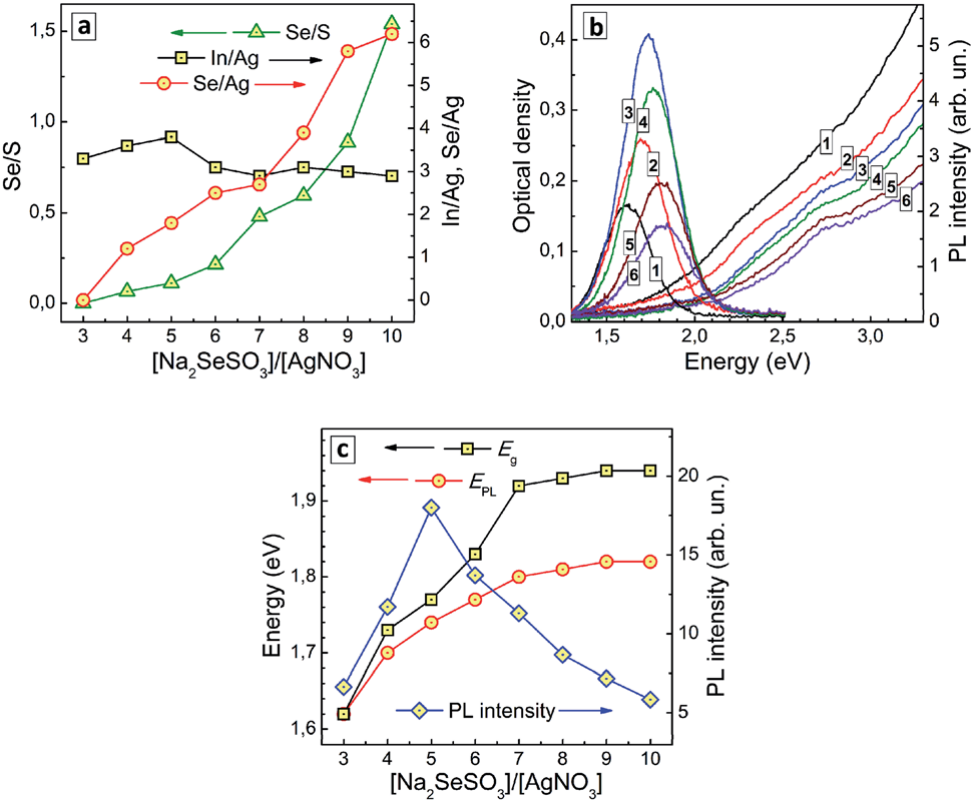
(a) Atomic ratios of Se to S (triangles), In to Ag (squares), and Se to Ag (circles) as functions of the nominal ratio of molar concentrations of Na_2_SeSO_3_ and AgNO_3_ during the QD synthesis. (b) Absorption and PL spectra of AISe QDs produced at a different [SS]/[Ag] ratios of 3 (curves 1), 4 (2), 5 (3), 7 (4), 8 (5), and 10 (6). (c) Bandgap (squares), PL band maximum energy (circles), and PL intensity (diamonds) as functions of the [SS]/[Ag] ratio. Lines connecting the points in (a) and (c) are guides for the eye only.

The composition of AISe QDs was indeed found to change with an increase in the nominal [SS]/[Ag^+^] ratio during the synthesis. In particular, the ratios of selenium-to-silver and selenium-to-sulfur were both found to increase monotonously with an increase in [SS]/[Ag^+^] ratio (Fig. 4a). No selenium was detected by XPS at [SS]/[Ag^+^] lower or equal to 4, while at a higher SS content the Se-to-Ag ratio increases almost linearly with the [SS]/[Ag^+^] ratio. At that, the ratio of indium-to-silver remains roughly the same indicating that an increase in the SS concentration does not change drastically the conditions of the QD formation. Inspection of this series of samples with high-resolution XPS shows that with no SS present the thermal treatment of colloidal solutions results in the formation of some amount of AIS QDs (ESI, Fig. S5a†), at intermediate [SS]/[Ag^+^] values AISe QDs are present along with free GSH ligands (ESI, Fig. S5b†), while at larger [SS]/[Ag^+^] values no free ligand can be found (ESI, Fig. S5c†). The absence of the ligand in the latter case results in incomplete coverage of the AISe QD surface which can be observed in XPS Se 3d spectral range as the presence of peaks assigned to undercoordinated selenide anions and Se^0^ (ESI, Fig. S5c†).

The absorption band edge of AISe QDs produced at different [SS]/[Ag^+^] ratios was found to shift to higher energies as the SS content is increased (Fig. 4b), the *E*^T^_g_ value growing from 1.62 eV at [SS]/[Ag^+^] = 4 to 1.94 eV at [SS]/[Ag^+^] = 10–11 (ESI, Table S3†). This effect can be explained by a higher rate of primary nucleation of AISe QDs at a larger rate of Se^2−^ release for higher SS concentrations resulting in smaller QDs.

The PL band maximum follows the position of the absorption band edge and shifts to higher energies with an increase of SS concentration – from 1.62 eV at [SS]/[Ag^+^] = 3 to 1.82 eV at [SS]/[Ag^+^] = 10 (Fig. 4b; ESI, Table S3†). At the same time, the PL intensity changes non-monotonously, increasing at lower SS concentration, reaching a maximum at [SS]/[Ag^+^] = 5 and decreasing at higher content of the selenosulfate anions (Fig. 4c).

The initial increase of the PL efficiency can be associated with a decrease of the AISe QD size. Deterioration of the emissive capability at [SS]/[Ag^+^] higher than 5 can originate from multiple factors, the most important one probably being an incomplete coverage of the QD surface with GSH ligands and formation of an indium selenide shell, which is then additionally oxidized as revealed by XPS. In all cases, the spectral width of the PL band remains roughly the same (in the range of 260–300 meV; ESI, Table S3†), despite the strong variation of the efficiency of PL emission. This fact additionally favours the self-trapped excitonic emission, rather than a defect-mediated (donor–acceptor pair) radiative recombination.

### Size selection series

Earlier we demonstrated the feasibility of size-selective fractionation of ensembles of AIS (AIS/ZnS) QDs^44,45^ and copper-doped AIS (AIS/ZnS) QDs^46^ by precipitating QDs with a dosaged addition of an anti-solvent followed by redispersion in DI water. A similar technique can also be applied to ensembles of GSH-capped AISe QDs as discussed below. The size-selective precipitation was performed using optimized AISe QDs with [In]/[Ag] = 4, [SS]/[Ag] = 5, and *t*_H_ = 60 min demonstrating the highest PL efficiency among all studied samples.

Totally, six fractions were separated from the original QD ensemble showing PL emission. Further addition of 2-propanol results in almost no precipitate, the fractions 7 and 8 exhibiting very low absorbance and no detectable PL signals.

**Fig. 5:**
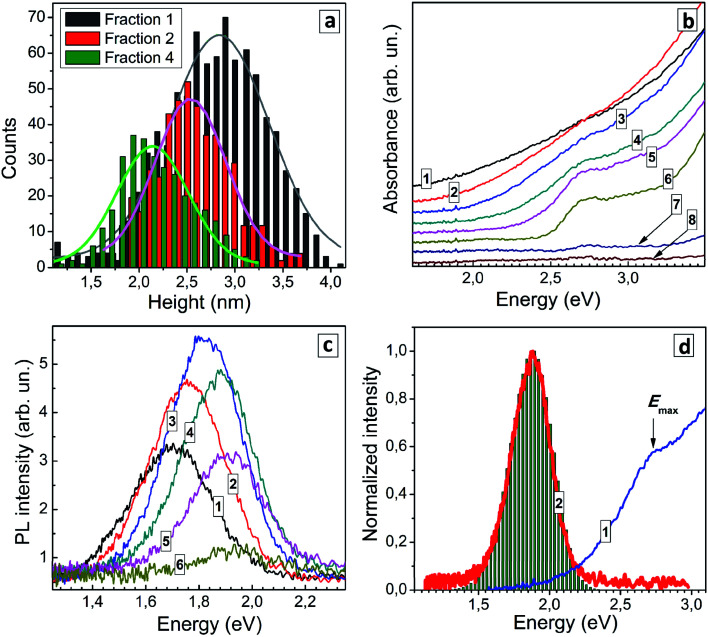
(a) Size distributions for size-selected AISe QDs from fractions 1, 2 and 4 derived from AFM images (solid curves represent fittings with Gaussian profiles). (b and c) Absorption spectra (b) and PL spectra (c) of size-selected AISe QDs (curve numbers correspond to fraction numbers). (d) Normalized absorption (curve 1) and PL (2) spectra of size-selected AISe QDs from fraction 4. Bars represent a modelled PL spectrum.

Three QD fractions were probed with AFM and showed a distinct difference in the average size, 2.8 nm for fraction 1, 2.5 nm for fraction 2, and 2.1 nm for fraction 4 (Fig. 5a) showing that the size-selection procedure was indeed successful. As the fraction number increases (and the average QD size decreases) the absorption band edge experiences a shift to higher energies and the peak at around 2.7 eV becomes more pronounced (Fig. 5b). Estimations made using Tauc plots showed that *E*^T^_g_ increases from 1.74 eV for fraction 1 up to 2.44 eV for fraction 6, while the position of absorption maximum shifts from 2.56 eV for fraction 1 to 2.72 for fraction 6 (ESI, Table S4†).

Earlier we have reported that a similar size-selection procedure applied to aqueous AIS QDs yields series of fractions of AIS QDs with a different average size but roughly the same composition.^43,44^ This finding allowed us to exclude possible composition variation as a major contributor to spectral changes observed in absorption and PL spectra of size-selected AIS QDs. We assumed a similar behavior for the present size-selected AISe QDs and validated this assumption by subjecting several QD fractions to the energy-dispersive X-ray spectroscopic (EDX) analysis (see ESI, Table S5†). The fractions 1, 2, and 4 showed similar atomic ratios of In to Ag (3.0–3.4), In to Se (0.8–1.0), and Se to S (1.1–1.2) indicating the similar QD composition in each of the probed size-selected fractions. Fraction 8 showed no signs of AISe with only In and S present (most probably from the residual In^III^–GSH complexes) which is in accordance with the absence of characteristic spectral features of AISe in the absorption spectrum of this fraction (Fig. 5b). The EDX results allow changes in the absorption and PL spectra of the size-selected AISe QDs to be assigned exclusively to variations of the average QD size.

The position of the PL band maximum was found to shift to higher energies as the fraction number increases (Fig. 5c), from 1.70 eV for fraction 1 to 1.94 eV for fraction 6 (ESI, Table S4†). The PL intensity increases in the first fractions, reaches a maximum for fraction 4 and then decreases for smaller AISe QDs (Fig. 5c). For all fractions the PL band shape can be fitted with a single Gaussian profile with the spectral band width varying between 275 and 305 meV. We can expect a narrowing of the size distribution for each next precipitated fraction of QDs (and Fig. 5a shows it to be the case for fractions 1 and 4). In view of this fact, the constant FWHM_PL_ in different fractions indicates that the PL band shape is not governed by size distribution of QDs but originates from inherent properties of the AISe QDs irrespective of their size. Such behavior is expected for the self-trapped excitonic (STE) mechanism of the broadband PL emission,^1,6,7^ when the PL band position shape are determined by the energy of interband electron transition (energy of purely electronic excited state), energy of phonons, and strength of electron–phonon coupling. As discussed previously for AIS (AIS/ZnS) QDs,^44,45^ in the frame of the STE model, the PL band is considered to be composed of a series *E*_*n*_ of phonon replicas of zero-phonon emission line (*E*_ZPL_), *E*_*n*_ = *E*_ZPL_ − *n* × *E*_ph_ (*E*_ph_ − phonon energy), while the intensity of each *E*_*n*_ contribution can be calculated as *I*_*n*_ = *S*^*n*^ × exp(−*S*)/*n*!, where *n* = 0, 1, 2, 3,… and *S* is the Huang–Rhys factor. The case of *n* = *S* corresponds to the maximum of the PL band, that is *E*_PL_ = *E*_ZPL_ − *S* × *E*_Ph_. The *E*_ZPL_ parameter can be derived from the position of a maximum in the absorption or PL excitation spectra.

Using the maximum energy of 2.69 eV and the energy of the most prominent AISe-related vibrational feature observed in Raman spectra (see discussion below), *E*_ph_ = 28 meV, we were able to reconstruct the shape of the PL band (compare curve 2 and bars in Fig. 5c) with a Huang–Rhys factor *S* = 29 resulting in a good correspondence between the experimental and calculated values of FWHM_PL_. We note that the presented results are of preliminary nature and a detailed study of the structure and composition of size-selected AISe QDs as well as their spectral PL parameters and time-resolved PL behavior is currently under way in our laboratory.

### Series on Zn concentration

Deposition of a wider-bandgap material shell on metal chalcogenide QDs is a conventional method for passivating surface defects and blocking interfacial electrons transfer, typically resulting in a higher PL efficiency. This approach is highly efficient also for aqueous ternary AIS and CIS QDs.^6,42–44^ At that, the In-based ternary QDs featured a remarkable diffusion of Zn^2+^ into the core that results in a “blue” shift of the absorption and PL bands and allows the optical properties of such core/shell CIS/ZnS and AIS/ZnS QDs to be modulated.^1,2,6^ As we showed previously,^44^ the modification of GSH-capped AIS core QDs (synthesized at [In]/[Ag] = 4) with a ZnS shell results in an increase of the PL quantum yield (QY) from 29 to 50% accompanied by a PL maximum shift from 2.01 to 2.07 eV (Table 1).

**Table 1:** Absorption and PL parameters of AIS (AIs/ZnS) and AISe (AISe/ZnS) QDs produced under optimal conditions

QD composition	PL maximum energy (eV/nm)	PL quantum yield (%)	PL FWHM (meV/nm)
AIS	2.01/620	29	320/100
AIS/ZnS	2.07/600	50	340/100
AISe	1.78/700	4	300/120
AISe/ZnS	1.91/650	12	350/120

A similar effect was observed here for GSH-capped AISe QDs. In this case, the modification of AISe QDs with ZnS results in a considerable growth of the PL QY – from 4% for “core” AISe QDs to 12% for “core/shell” AISe/ZnS (Table 1, QDs produced at an optimal AISe-to-ZnS ratio, see discussion below).

**Fig. 6:**
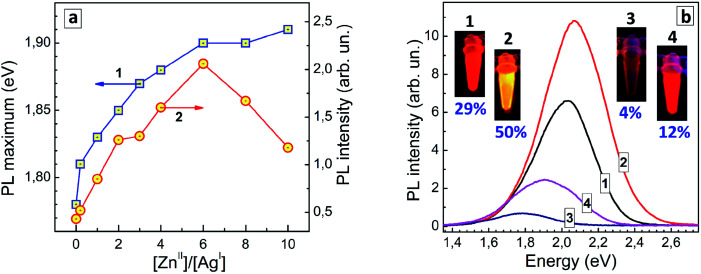
(a) PL band maximum energy (curve 1) and PL intensity (curve 2) of AISe and AISe/ZnS QDs synthesized at different molar [Zn]/[Ag] ratios. Lines connecting the experimental points are guides to the eye only. (b) PL spectra, photographs and PL QYs of four representative colloidal solutions of GSH-capped QDs – AIS (curve and photograph 1), AIS/ZnS (2), AISe (3), and AISe/ZnS (4).

The shift of the PL maximum observed for AISe/ZnS with respect to AISe QDs, 0.13 eV, is more than twice larger than that observed for the AIS-based counterparts (0.06 eV). The spectral shift induced by Zn^2+^ diffusion into the AISe core material depends on the nominal ratio of Zn(ii) to Ag(i) species during the formation of such core/shell QDs (Fig. 6a, curve 1) providing an additional method for fine tuning of the PL properties of AISe QDs. The PL intensity grows with increasing Zn-to-Ag ratio, reaching a maximum at [Zn]/[Ag] = 6 and decreasing at a higher zinc content (Fig. 6a, curve 2).

The latter decrease is observed on the background of a tiny increment of the PL maximum energy (compare curves 1 and 2 in Fig. 6a) allowing a more massive Zn doping to be excluded as a viable reason for the PL quenching at [Zn]/[Ag] > 6. Most probably, over-abundance of Zn^II^ species results in a partial agglomeration of AISe/ZnS QDs (*via* interparticle binding, surface re-charging, *etc.*) and/or desorption of GSH ligands suppressing the radiative recombination.

Fig. 6b shows PL spectra of representative AIS, AIS/ZnS, AISe, and AISe/ZnS QD colloids with corresponding photographs taken under UV illumination (350–370 nm). The PL QY of 12% was the highest achieved in the present work showing a large room for further improvement of the synthetic procedure and control over the emission properties of AISe QDs.

### Vibrational properties of AISe QDs probed by Raman spectroscopy

**Fig. 7:**
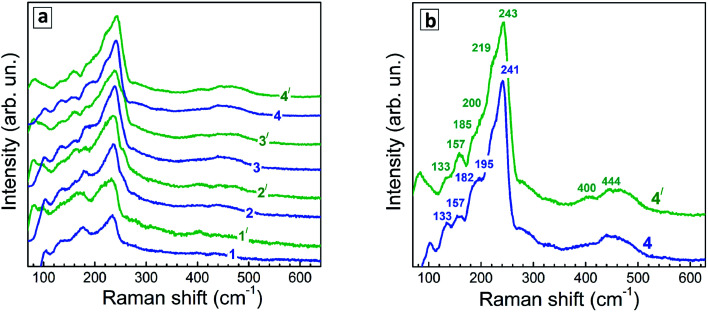
(a) Representative low-temperature (80 K) Raman spectra of Ag–In–S samples produced at different duration of the thermal treatment – 0 (1), 10 min (2), 45 min (3), and 240 min (4). Spectra are registered at 488 nm (spectra 1–4) and 515 nm (spectra 1′- 4′) excitations. (b) spectra of the QDs heated for 240 min, shown in more detail.

Representative Raman spectra of Ag–In–S QDs are shown in Fig. 7a for the series of QD samples obtained at different duration of thermal treatment.

**Fig. 8:**
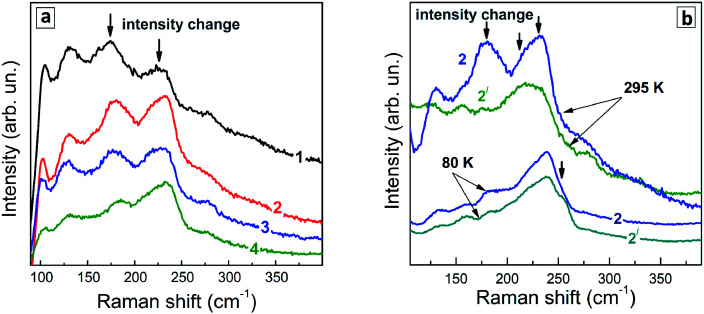
(a) Representative room-temperature Raman spectra (*λ*_exc_ = 488 nm) of (a) a series of Ag–In–S QDs obtained at different duration of a thermal treatment – 0 (1), 45 (2), 100 (3), and 240 min (4). (b) Spectra of sample with *t*_H_ = 45 min measured at *λ*_exc_ = 488 nm (curves 2) and 515 nm (curves 2′) at room temperature (295 K) and *T* = 80 K.

Even though the low-temperature Raman spectra (Fig. 7b) expectedly have a better signal-to-noise ratio and allow more peaks to be resolved due to smaller homogeneous broadening of the phonon peaks, there are some spectral trends that are better seen in the room temperature spectra. In particular, the redistribution of the scattering intensity within the series corresponding to different duration of the thermal treatment (Fig. 8a).

Phonon spectra of Ag–In–Se have been poorly studied and understood so far. This is contrary to related compounds like CuInS_2_ and CuInSe_2_,^57–59^ phonon spectra of which were addressed theoretically^60–62^ and experimentally by Raman^57,58^ and IR spectroscopies,^60^ including QDs.^61^

The strongest Raman active mode of tetragonal (chalcopyrite) CuInSe_2_ is the A_1_-symmetry mode at 175 cm^−1^, observed both in bulk samples^57,58^ and QDs.^63^ Besides, the so-called CuAu structural modification can exist in both CuInSe_2_ and CuInS_2_, predominantly in Cu-poor samples, which has a characteristic A_1_ mode at about 185 cm^−1^.^64^ This fact needs to be kept in mind when analyzing the phonon spectra of the present Ag-poor Ag–In–Se QDs.

The structural similarity among various I–III–VI compounds can be expected to lead to analogous features also in their vibrational spectra. The A_1_(CuAu) mode in the case of CuInS_2_ is observed at about 10 cm^−1^ higher frequency compared to the A_1_ mode of the chalcopyrite structure, similarly to CuInSe_2_.

We recently observed evidences of an analogous structural phase in ultrasmall Ag-poor Ag–In–S QDs.^52^ Note that, despite common referring to the samples as CuInSe_2_ or CuInS_2_, the elemental composition of the samples is not always corresponding to this stoichiometry. On the contrary, in the case of small colloidal QDs the elemental ratios of the samples can be tuned in the whole range of Cu : In or Ag : In ratios.^42–47^ At the same time, the vibrational (Raman or IR) phonon frequencies were reported so far only for a few of particular structural modifications of a fixed/certain stoichiometry. Thus, for the CuIn_3_Se_5_ compound, the main (A_1_) Raman mode was reported at about 150 cm^−1^,^65^ which finds no matching in the Raman spectra of the present AISe QDs. In somewhat better agreement are the frequencies calculated for IR-active phonons in AgIn_3_Se_5_;^66^ however, a different set of vibrational modes is generally expected in Raman and IR. Furthermore, so-called “ordered vacancy compounds (OVCs)”, practically indistinguishable from chalcopyrite CISe by XRD, were identified by means of Raman spectroscopy,^67^ in analogy to the OVC modification of CIS, described earlier.^57,58^ For the OVC CISe structure, relatively broad, as compared to the A_1_ modes at 175 or 185 cm^−1^, Raman bands at about 120 cm^−1^ and 250 cm^−1^ were reported.^67^

The Raman spectra of the Ag–In–Se QDs studied in this work can be well described by the contribution of the latter four features (Fig. 8a), with the 175 and 185 cm^−1^ most likely both contributing to the broad experimental feature centered around 175 cm^−1^. The agreement can be regarded as especially good in view of the small size of our QDs, not exceeding 3 nm, and their notable non-stoichiometry with respect to the stoichiometric AgInS_2_ compound. The relative intensity of the 175 cm^−1^ chalcopyrite feature gets weaker at longer thermal treatment of the QDs (Fig. 7a), or when excited with 488 nm instead of 515 nm laser line (Fig. 7a and b). The first trend is in good agreement with the more Ag-poor composition of the QDs subject to longer thermal treatment (as concluded from the XPS results discussed above), because for more Ag-deficient lattice the relative intensity of the OVC peak (at 185 cm^−1^) is expected to get higher. The effect of the energy of the exciting quantum (∼*λ*_exc_) is even more interesting, because it may directly reflect the noticeable difference in electronic band structure of chalcopyrite, CuAu, and OVC modifications, a fact that could be only hypothesized so far. Multi-*λ*_exc_ Raman spectroscopy has already proved to be able to probe selectively different compounds or phases contained within the same individual QDs in the ensemble.^54^

On the other hand, the heating-induced changes of the Raman spectra of AISe QDs are generally similar to those we previously observed for CuInS_2_,^52^ and attributed to increased relative intensity of the highest-frequency LO modes, situated at 340–350 cm^−1^ for CuInS_2_ (ref. 61) and, presumably, at 230–240 cm^−1^ for present AISe QDs. This assumption is in good qualitative agreement with the position of these modes obtained from the (only reported) experimental study of IR phonons in AgInSe_2_ (ref. 68) and their theoretical calculations.^69^ It is important to note that the resonant enhancement of the LO modes was shown to change the spectrum, for example, that of CuInS_2_, dramatically, with the A_1_ mode being not dominant in the spectrum anymore. A similar situation may not be excluded in our case of AISe QDs, because their Raman spectra show clear indications of resonant behavior as well, in particular the above discussed different relative intensity of the peaks when excited with different *λ*_exc_ = 488 nm and 514.7 nm (see Fig. 7). This may explain the A_1_ mode at 175 cm^−1^ being not the strongest peak, and observing instead maximum scattering intensity in the range of 220–240 cm^−1^ (in the probable range of LO phonons). Note, however, that in the case of non-stoichiometric Cu–In–S QDs,^52^ the trend in A_1_/LO intensity ratio with *λ*_exc_ was opposite to the one observed for bulk or stoichiometric QD samples.^61^ Based on this fact and the present dependence of the Raman spectra on *λ*_exc_ (Fig. 7), we can conclude that the Raman spectra of the strongly non-stoichiometric QDs are determined not only by the lattice structure but even more strongly by the details of the electronic band structure and concomitant resonances with *λ*_exc_, as well as by the details of the electron–phonon coupling.

A certain resemblance of our AISe QDs spectra to those of different structural modifications of In_2_Se_3_ (ref. 70) is not surprising in view of the notable Ag deficiency of the present QDs. However, none of the In_2_Se_3_ (or InSe) spectra matches our QD spectra close enough.

## Conclusions

We introduced a protocol for a direct aqueous synthesis of luminescent 2–3 nm AISe QDs capped by multifunctional and biocompatible GSH ligands which benefits from using Na_2_SeSO_3_ as a stable precursor releasing Se^2−^ anions *in situ* in a readily controllable way with no additional Se-containing by-products. As in the previously studied case of aqueous AIS–GSH QDs, the AISe colloids emit broadband PL with its efficiency depending in a non-linear way on basic synthesis parameters, including nominal ratios of silver-to-indium and selenosulfate-to-silver, as well as on the duration of the post-synthesis thermal treatment at 96–98 °C and the presence of a ZnS shell on AISe QDs. An optimization of these parameters allowed conditions to be identified for the formation of the most emissive AISe QDs revealing PL QYs of 4% and 12% for core AISe and core/shell AISe/ZnS QDs. Similar to AIS–GSH QDs, the as-prepared ensembles of AISe–GSH QDs can be subjected to a size-selective precipitation/redispersion allowing a series of size-selected AISe QDs to be produced with distinctly different positions of absorption and PL bands.

For all sizes and compositions the PL is emitted in broad bands with the maximum variating from around 1.65 eV (750 nm) to 1.90 eV (650 nm) and a spectral width of around 300 meV (100–120 nm) depending on the average size and composition. The shape and position of PL bands is interpreted in terms of a model of radiative recombination of a self-trapped exciton.

We probed the vibrational properties of GSH-capped non-stoichiometric AISe QDs by temperature-dependent Raman spectroscopy. The observed bands do not match exactly with phonon patterns of known structural modifications of AgInSe_2_, AgIn_5_Se_8_, AgIn_3_Se_5_, and CuAu or OVC phases. Nevertheless, their frequencies coincide in terms of the spectral range, which corroborates their attribution to the “bulk-like” lattice phonons of AIS. We can conclude, therefore, that the observed phonon spectra are characteristic for small and Ag-deficient tetragonal Ag–In–Se QDs. The ability of this crystal structure to support “bulk-like” vibrations is noteworthy. This phenomenon can be used for further better understanding of the optical and other properties of this relatively new sort of QDs.

The present protocol can potentially be extended for the synthesis of other aqueous QD species, including Cu–In–Se, (Cu,Ag)–In–Se and (Cu,Ag)–In-(S, Se) QDs as well as more complex compositions produced by diffusing these QDs with Zn^2+^ and (possible) other cations.

## Conflicts of interest

The authors declare no conflicts of interests.

## Supplementary Material

RA-010-D0RA07706B-s001
