# Supplementary material for: Ultra-small aqueous glutathione-capped Ag–In–Se quantum dots: luminescence and vibrational properties
Source: RSC Adv. 2020 Nov 19;10(69):42178–93. doi: 10.1039/d0ra07706b (PMC9057868; doi:10.1039/d0ra07706b)
Supplement: RA-010-D0RA07706B-s001 [file RA-010-D0RA07706B-s001.pdf]

Electronic Supplementary Material (ESI) for RSC Advances.
This journal is © The Royal Society of Chemistry 2020

Supporting Information

Ultra-Small Aqueous Glutathione-Capped Ag-In-Se Quantum Dots: Luminescence and Vibrational Properties

Oleksandra Raievska[1-3], Oleksandr Stroyuk[3,4*],
Volodymyr Dzhagan[5,6], Dmytro Solonenko[1,2], Dietrich R.T. Zahn[1,2*]

[1]*Semiconductor Physics, Chemnitz University of Technology, D-09107 Chemnitz, Germany*
[2]*Center for Materials, Architectures, and Integration of Nanomembranes (MAIN), Chemnitz University of Technology, D-09107 Chemnitz, Germany*
[3]*L.V. Pysarzhevsky Institute of Physical Chemistry, Nat. Acad. of Science of Ukraine, 03028 Kyiv, Ukraine*
[4]*Forschungszentrum Jülich GmbH, Helmholtz-Institut Erlangen Nürnberg für Erneuerbare Energien (HI ERN), 91058 Erlangen, Germany*
[5]*V. Lashkaryov Institute of Semiconductors Physics, National Academy of Sciences of Ukraine, Kyiv, Ukraine*
[6]*Taras Shevchenko National University , 01601 Kyiv, Ukraine*

Authors for correspondence:

[*]Dr. Oleksandr Stroyuk, Forschungszentrum Jülich GmbH, Helmholtz-Institut Erlangen Nürnberg für Erneuerbare Energien (HI ERN), Immerwahrstr. 2, 91058 Erlangen, Germany; *e-mail*: o.stroyuk@fz-juelich.de, alstroyuk@ukr.net

[*]Prof. Dietrich R.T. Zahn, Semiconductor Physics, Chemnitz University of Technology, Reichenhainer Straße 70, 09126 Chemnitz, Germany; *e-mail*: zahn@physik.tu-chemnitz.de

Table S1. Absorption and PL parameters of AISe QDs synthesized at different [In]/[Ag] ratios.

[In]/[Ag]^{l}	E_{PL} (eV)	FWHM$_{PL}$ (meV)	I_{PL} (arb. un)	E_g^T (eV)	E_{max} (eV)
20.0	1.94	360	0.5	-	2.78
10.0	1.91	335	2.6	2.15	2.72
6.7	1.83	340	6.4	1.97	2.69
5.0	1.75	343	7.1	1.96	2.68
4.4	1.71	345	7.1	1.84	2.66
4.0	1.70	350	6.7	1.81	2.65
3.6	1.69	355	5.8	1.78	2.65
3.3	1.69	355	5.3	1.72	2.64
2.7	1.69	360	4.2	1.72	2.63

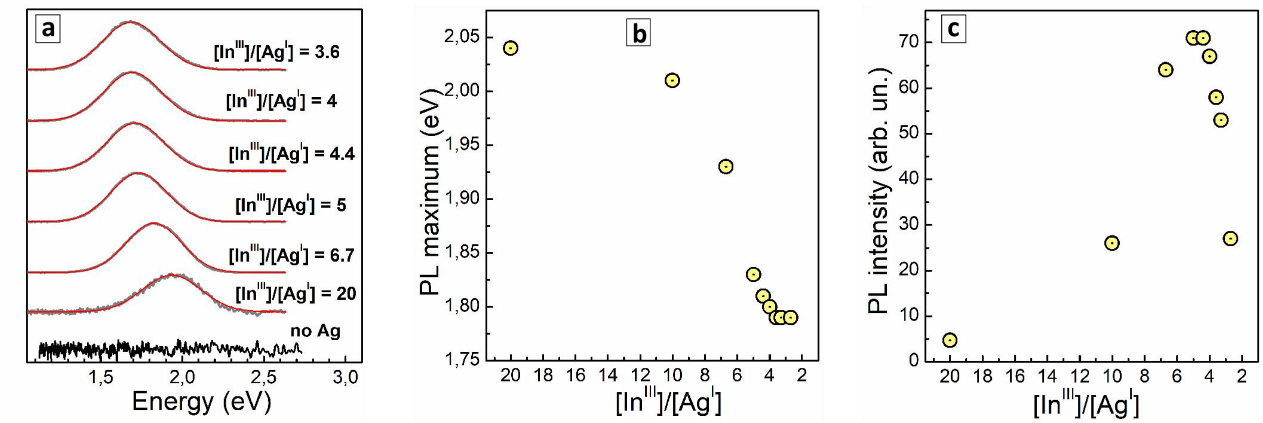

Figure S1. (a) Normalized PL spectra (gray lines) of AISe QDs produced at different [In]/[Ag] ratios. Red lines show fitting of the spectra with single Gauss profiles. (b, c) PL band maximum position (b) and integral PL intensity (c) of AISe QDs produced at different [In]/[Ag] ratios.

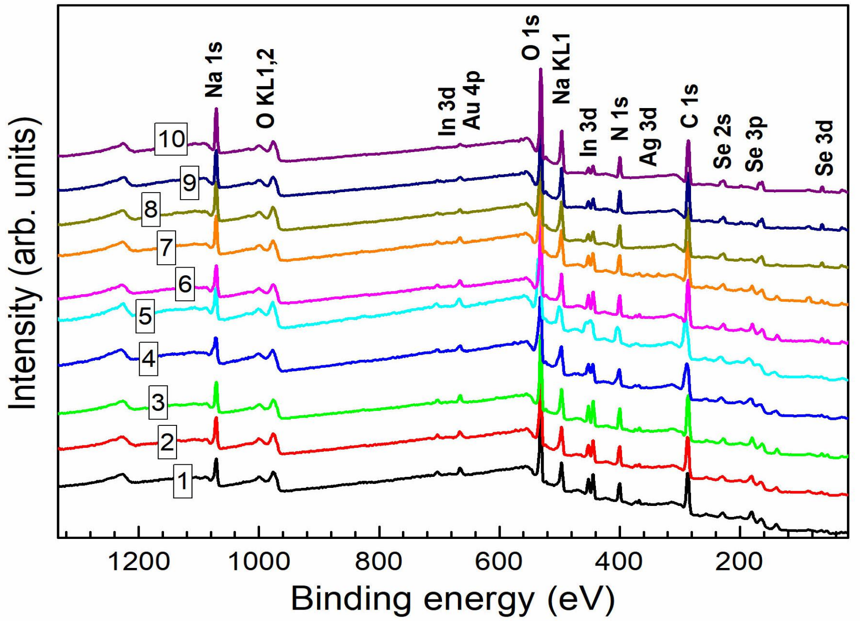

Figure S2. Survey X-ray photoelectron spectra of AISe QDs subjected to no heating (curve 1) and to a thermal treatment at 96-98°C for 2 min (curve 2), 10 min (3), 30 min (4), 45 min (5), 60 min (6), 100 min (7), 130 min (8), 180 min (9), and 240 min (10).

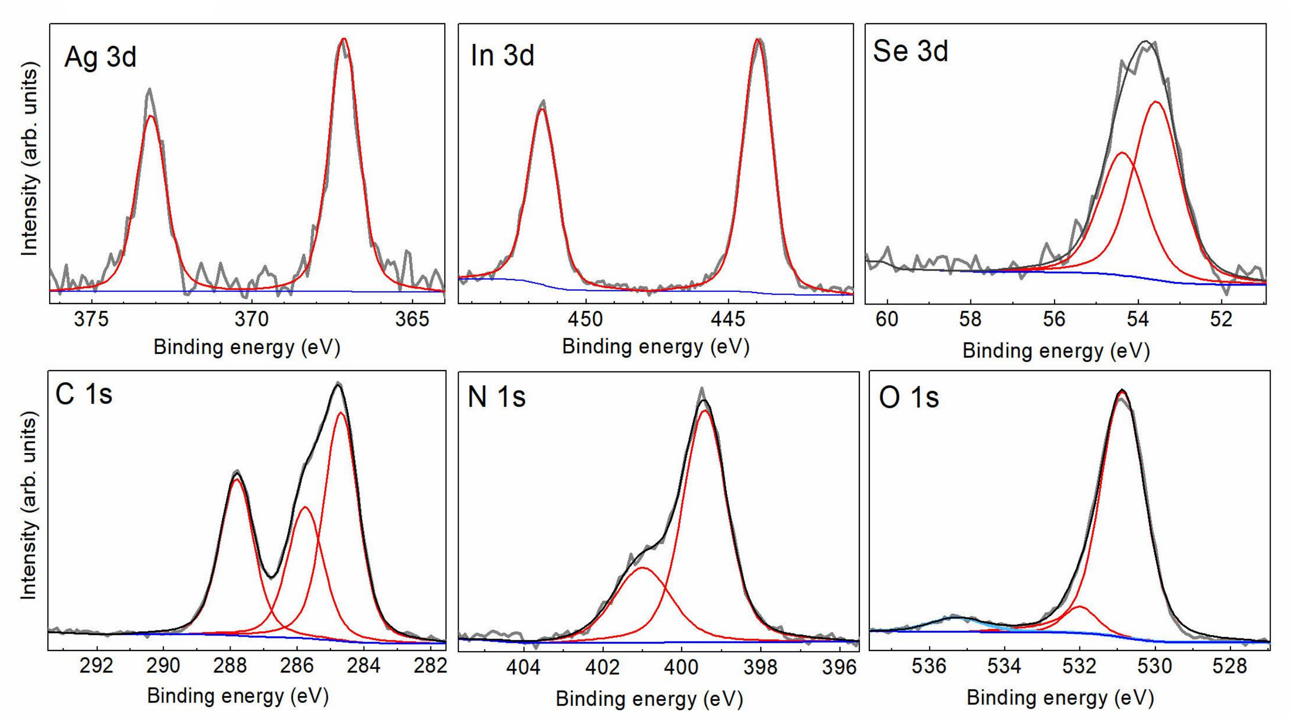

Figure S3. High-resolution X-ray photoelectron spectra in the ranges of Ag3d, In3d, Se3d, C1s, N1s, and O1s electron binding energies for AISe QDs produced at Ag-to-In ratio of 1:4 and 60-min thermal treatment at 96-98°C. Gray lines represent experimental data, red lines - fitting with Gauss profiles, black lines - enveloping contours, blue lines – baselines.

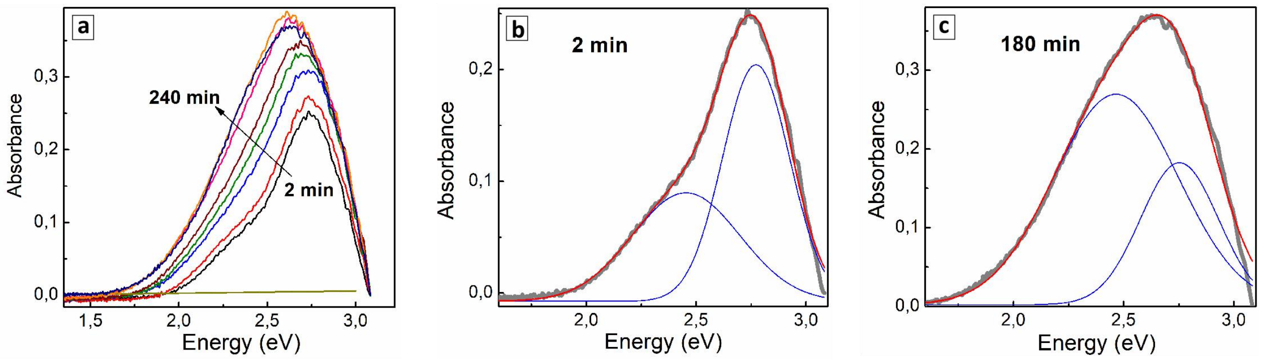

Figure S4. (a) Differential absorption spectra of AISe QDs produced by subtracting spectrum for t = 0 min from spectra of colloidal solutions heated for 2-240 min. (b,c) exemplify fitting of differential absorption spectra of AISe QDs heated for 2 min (b) and 180 min (c) with combinations of two Gauss profiles.

Table S2. PL parameters of AISe QDs produced with a different duration of thermal treatment

**Heating duration**	E_{PL} (eV)	$FWHM_{PL}$ (meV)	I_{PL} (arb. un.)
0	1.99	260	0.2
2	1.95	290	0.4
10	1.90	295	1.4
30	1.86	295	3.9
45	1.84	300	4.4
60	1.81	300	5.9
100	1.77	310	5.0
130	1.73	310	2.5
180	1.68	300	1.8
240	1.66	290	0.7

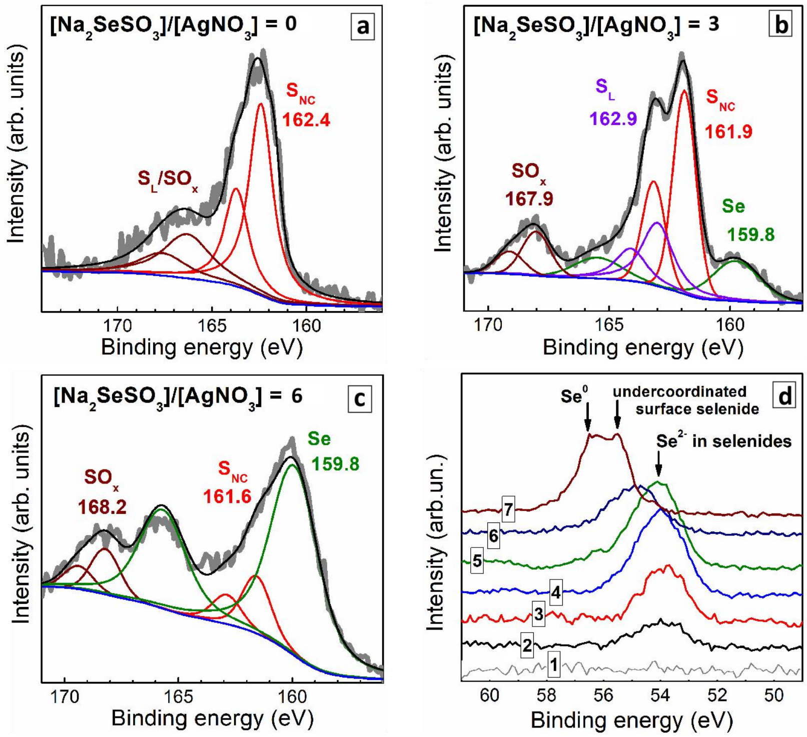

Figure S4. (a-c) High resolution X-ray photoelectron spectra (gray) in the range of S2p/Se3p binding energies for QDs produced at different $[Na_2SeSO_3]/[AgNO_3]$ ratios. Multicolored lines represent the best fit of the experimental spectra with combinations of Gauss profiles, black is the total envelope profile, and blue is the baseline. (d) XPS spectra in the Se3d binding energy range of AISe QDs synthesized at $[Na_2SeSO_3]/[AgNO_3]$ = 0 (curve 1), 1 (2), 2 (3), 3 (4), 4 (5), 5 (6), and 6 (curve 7).

Table S3. Absorption and PL parameters of AISe QDs produced at different ratios of [Na_2SeSO_3] to [$AgNO_3$]

[Na_2SeSO_3]/[$AgNO_3$]	E_g^T (eV)	E_{PL} (eV)	FWHM$_{PL}$ (meV)
3	1.62	1.62	260
4	1.74	1.70	280
5	1.77	1.74	290
6	1.83	1.77	290
7	1.94	1.80	295
8	1.93	1.81	295
9	1.94	1.82	295
10	1.94	1.82	300

Table S4. Absorption and PL parameters of size-selected AISe QDs

Fraction number	E_g^T (eV)	E_{max} (eV)	E_{PL} (eV)	FWHM$_{PL}$ (meV)	I_{PL} (arb. un.)
1	1.74	2.56	1.70	305	1.8
2	1.82	2.64	1.76	295	7.9
3	1.90	2.68	1.82	280	13.7
4	2.09	2.69	1.87	275	17.3
5	2.21	2.70	1.91	275	14.8
6	2.44	2.72	1.94	300	10.2

Table S5. Summary of the results fo EDX analysis for fractions 1, 2, 4, and 8 of GSH-stabilized size-selected AISe QDs

Fraction	Atomic fraction, %				Atomic ratio		
	Ag	In	Se	S	In/Ag	In/Se	Se/S
1	2.3	7.2	7.9	6.2	3.13	0.91	1.27
	2.5	7.4	7.9	6.5	2.96	0.94	1.22
	2.5	7.6	7.8	6.6	3.04	0.97	1.18
	2.4	7.6	7.8	6.7	3.17	0.97	1.16
**Average**	**2.5**	**7.5**	**7.9**	**6.5**	**3.10**	**0.95**	**1.21**
2	2.7	8.3	10.3	9.2	3.07	0.81	1.12
	2.7	8.2	10.2	9.0	3.04	0.80	1.13
	2.7	8.1	10.0	9.1	3.00	0.81	1.10
**Average**	**2.7**	**8.2**	**10.2**	**9.1**	**3.04**	**0.81**	**1.12**
4	1.7	5.7	6.3	5.4	3.35	0.90	1.17
	1.5	5.2	5.7	4.9	3.47	0.91	1.16
	1.7	5.8	6.1	5.8	3.41	0.95	1.05
**Average**	**1.5**	**5.6**	**6.0**	**5.4**	**3.41**	**0.92**	**1.13**
8	-	0.4	0.1	7.6	-	0.25	0.01
	-	0.4	0.1	7.4	-	0.25	0.01
**Average**	-	**0.4**	**0.1**	**7.5**	-	**0.25**	**0.01**

Notes: each sample was probed in several different spots and the atomic fractions and ratios averaged. EDX analysis was performed on a JEOL JSM-7610F Schottky field emission scanning electron microscope operating under 10 kV acceleration voltage. The samples were prepared by drop-casting the QD colloids onto FTO glass followed by drying in vacuum.
